# Use of fluorescent ANTS to examine the BBB-permeability of polysaccharide

**DOI:** 10.1016/j.mex.2015.03.006

**Published:** 2015-03-18

**Authors:** Kevin Christopher, Vishruti Makani, Wesley Judy, Erica Lee, Nicolas Chiaia, Dong Shik Kim, Joshua Park

**Affiliations:** a Department of Neurosciences, University of Toledo, College of Medicine and Life Science, Toledo, OH 43614, USA; b Department of Chemical Engineering, University of Toledo, College of Engineering, Toledo, OH 43607, USA

**Keywords:** Quantifying the BBB permeability of fluorescent-tagged polysachharide in animals, Blood brain barrier (BBB)-permeability, Polysaccharide tracking, ANTS labeling

## Abstract

Recently, some polysaccharides showed therapeutic potentials for the treatment of neurodegenerative diseases while the most important property, their permeability to the blood brain barrier (BBB) that sheathes the brain and spinal cord, is not yet determined. The determination has been delayed by the difficulty in tracking a target polysaccharide among endogenous polysaccharides in animal. We developed an easy way to examine the BBB-permeability and, possibly, tissue distribution of a target polysaccharide in animal. We tagged a polysaccharide with fluorescent 8-aminonaphthalene-1,3,6-trisulfonic acid disodium salt (ANTS) for tracking. We also developed a simple method to separate ANTS-tagged polysaccharide from unconjugated free ANTS using 75% ethanol. After ANTS-polysaccharide was intra-nasally administered into animals, we could quantify the amounts of ANTS-polysaccharide in the brain and the serum by fluorocytometry. We could also separate free ANTS-polysaccharide from serum proteins using trichloroacetic acid (TCA) and 75% ethanol. Our method will help to track a polysaccharide in animal easily.

• ANTS-labeling is less tedious than but as powerful as radiolabeling for tracking a target polysaccharide in animal.
• Our simple method can separate structurally intact ANTS-polysaccharide from animal serum and tissues.
• This method is good for the fluorometry-based measurement of ANTS-conjugated macromolecules in tissues.

## Graphical abstract

**Figure:**
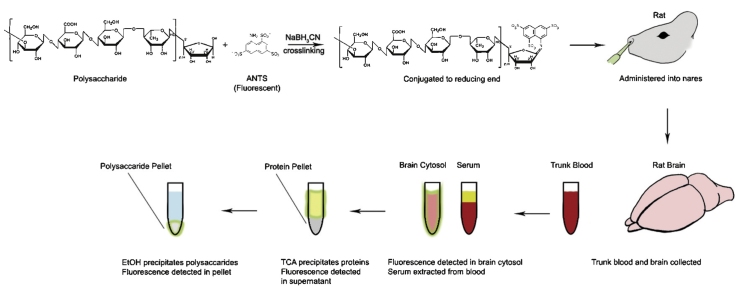

## 1 Method details

This is a simple and effective method developed to track an exogenous polysaccharide among endogenous polysaccharides in animals. Instead of using complicated methods and equipment, a fluorescent tag, ANTS, can be used to label, track, and quantify a target polysaccharide in animal. 75% ethanol can be used to examine the structural intactness of ANTS-polysaccharide in animal sample. TCA and 75% ethanol can be used to separate protein-free ANTS-polysaccharides from those bound to proteins.

## 2 Recommended equipment

• SpectraMax M5 plate reader (Molecular Devices, Sunnyvale, CA)
• VERSA max plate reader (Molecular Devices)
• SoftMax Pro 5.2. (Molecular Devices)
• Microcentrifuge
• Ultracentrifuge (WX Ultra 80, Thermo Scientific, Waltham, MA)
• AH650 rotor and buckets (Thermo Scientific)

## 3 Conjugation of ANTS to polysaccharide

A 4.7 kD cleavage product of gellan gum (named midi-GAGR: digestion by *α*(1 → 3) glycosidase) was tagged with ANTS and examined regarding its BBB-permeability.

1. To conjugate ANTS (Molecular Probes, Eugene, OR) to midi-GAGR, 45 μL of 7.4 mM midi-GAGR was mixed with 750 μL of 0.02 M ANTS (7.6 mg ANTS in 890 μL of acetic acid/water (3/17, v/v)), which gives the final ratio of 1:400 (polysaccharide:ANTS) that is optimal for high-efficiency conjugation between polysaccharide and ANTS [1].
2. The mixture was briefly vortexed and incubated in an 80 °C water bath for 30 min.
3. The mixture was then added with 375 μL of 1 M NaCNBH_3_ (Sigma–Aldrich, St. Louis, MO), briefly vortexed, and incubated in an 80 °C water bath for 90 min. Although 37 °C can be also used for 15-h conjugation [2], 80 °C was used to shorten conjugation time [1].
4. The mixture was split into 250-μL aliquots, each of which was mixed with 750 μL of 100% pure ethanol to make a final concentration of 75% ethanol.
5. The mixture was briefly vortexed, incubated at −80 °C for 30 min, and centrifuged at 3000 × *g* for 30 min to pellet ANTS-tagged midi-GAGR.
6. In order to remove free ANTS that might be trapped in ANTS-midi-GAGR pellet, the pellet was washed with 400 μL of 75% ethanol to dissolve ANTS trapped in the pellet. After the pellet of ANTS-midi-GAGR was resuspended in 400 μL of 75% ethanol by pipetting, it was re-precipitated at 3000 × *g* for 10 min. This step was repeated three times. The final pellet was resuspended in 50 μL of sterile de-ionized water.

(*Note*: centrifugation at 15,700 ×* g* was also used for ethanol precipitation of ANTS-tagged polysaccharide. However, it resulted in precipitation of free ANTS. Therefore, we decreased the speed of centrifugation to 3000 ×* g* and could prevent the precipitation of free ANTS while still precipitating the similar amount of polysaccharide to that after the centrifugation at 15700 ×* g*. We also used 70% ethanol instead of 75% to reduce the precipitation of free ANTS that might be trapped in ANTS-polysaccharide pellet. Washes with 70% ethanol precipitation decreased the amount of ANTS at the pellet; however, there was also a noticeable loss of polysaccharide at the pellet after each wash. Thus, 75% ethanol was an optimal concentration of ethanol to precipitate the maximal amount of polysaccharide and the minimal amount of free ANTS.)

## 4 Calculation of the conjugation ratio of ANTS to polysaccharide in ANTS-polysaccharide

We measured the amounts of ANTS and midi-GAGR in ANTS-polysaccharide conjugate by fluorometry and colorimetry, respectively, to calculate the ratio of ANTS to midi-GAGR in the conjugate.

### 4.1 For fluorometry,

1. ANTS-polysaccharide pellet was resuspended and diluted in 50 μL of fresh water.
2. The dilutions were placed in the wells of a 96-well black-wall plate.
3. The emission fluorescence signals (excitation at 350 nm, emission at 520 nm; relative fluorescence units (RFUs)) of the dilutions were measured using SpectraMax M5 plate reader and SoftMax Pro 5.2.
4. We generated a standard curve of ANTS using 0, 0.1, 0.3, 1, 3, and 10 mM ANTS to calculate the concentrations of ANTS in the samples.

(*Note*: given that the fluorescence intensity of fluorophore is not affected by the conjugation to polysaccharide [1], we do not expect that conjugation of midi-GAGR to ANTS changes the RFU of ANTS. Nonetheless, we could not use midi-GAGR-conjugated ANTS to generate a standard curve because free ANTS that was removed from ANTS-midi-GAGR during multiple EtOH washes after conjugation reaction was not accurately quantifiable to obtain the exact concentration of ANTS in the final pellet of ANTS-midi-GAGR.)

### 4.2 For colorimetry,

We used a phenol-sulfuric acid method modified from a previous one [3] to measure the concentration of the polysaccharide, midi-GAGR, in ANTS-midi-GAGR conjugate.

1. ANTS-polysaccharide pellet was resuspended and diluted in 50 μL of fresh water. The dilutions were placed in the wells of a 96-well clear-wall plate.
2. Each sample was added with 150 μL of concentrated H_2_SO_4_ and then 30 μL of 5% phenol (88% phenol liquefied USP (University of Toledo Medical Center, Toledo, OH) diluted in distilled water).
3. The top of the plate was covered with a plate sealer and heated at 95 °C for 5 min.
4. Using VERSA max plate reader and SoftMax Pro 5.4, the absorbance at 490 nm of each well was measured.
5. We generated a standard curve of midi-GAGR using 0, 0.0074, 0.074, 0.74, and 7.4 mM midi-GAGR to calculate the concentrations of midi-GAGR in the samples.

### 4.3 Calculation of the ratio of ANTS to midi-GAGR in the conjugate

**Fig. 1:**
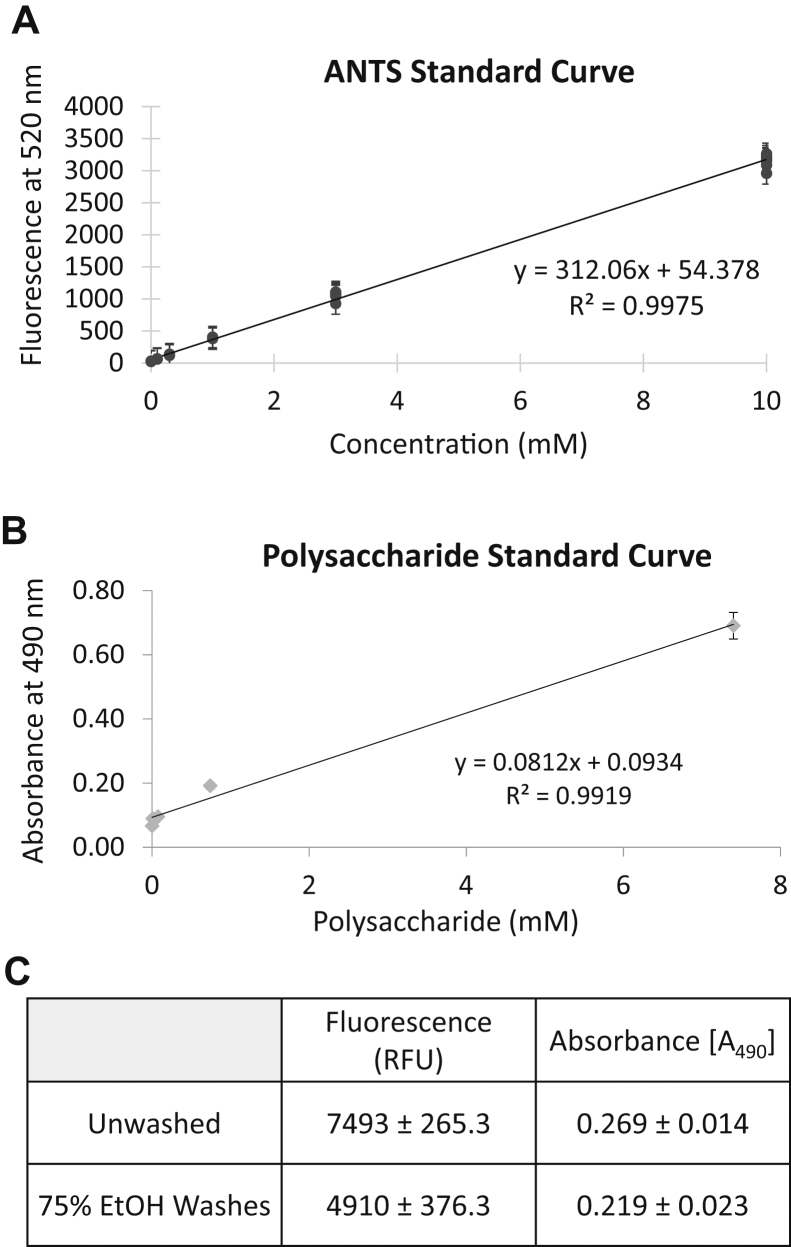
(A) The standard curve of emissions at 520 nm (relative fluorescence unit: RFU) of ANTS at 0, 0.1, 0.3, 1, 3, and 10 mM (*n* = 5). (B) The standard curve of absorbances at 490 nm for 0, 7.4, and 74 nM, 0.74 and 7.4 mM of midi-GAGR (*n* = 3). (C) Fluorescent and colorimetric values of ANTS-midi-GAGR before and after three 75% ethanol washes (Mean ± Std. Error, *n* = 5).

1. We made the standard curve of ANTS using the RFUs of 0, 0.1, 0.3, 1, 3, and 10 mM free ANTS to quantify the concentrations of ANTS in the samples. Fig. 1A shows the standard curve of ANTS (*R*^2^ = 0.9975) that was generated on the basis of three different measurements.
2. We also made the standard curve of midi-GAGR using the absorbances at 490 nm for 0, 0.0074, 0.074, 0.74, and 7.4 mM free midi-GAGR. Fig. 1B shows the standard curve of midi-GAGR (*R*^2^ = 0.9919).
3. According to the standard curves, the RFU of ANTS-polysaccharide in the pellet before wash was ∼7493 and its absorbance at 490 nm was ∼0.269 (Fig. 1C). After three washes, the mean RFU of ANTS-polysaccharide was significantly reduced to ∼4910 while the absorbance at 490 nm was only slightly reduced to 0.219. The values of RFU and absorbance at 490 nm were not further decreased by more washes after the third wash, suggesting that most of the loosely-associated free ANTS was removed from the pellet of ANTS-midi-GAGR.
4. According to the standard curves, the final pellet contained 16.18 mM ANTS and 1.55 mM midi-GAGR, which give the ratio of about 10:1 for ANTS to midi-GAGR.

(*Note*: one ANTS was supposed to be conjugated to one reducing end of a polysaccharide, thus yielding the ratio of 1:1 for ANTS to midi-GAGR. However, more ANTS appeared to be conjugated to other hydroxyl groups on midi-GAGR, yielding the 10:1 ratio for ANTS to midi-GAGR. We also tried to label polysaccharide with ANTS using EDC (1-ethyl-3-(3-dimethylaminopropyl)carbodiimide) that should conjugate the amino group of ANTS to the carboxyl group of glucuronic acid of midi-GAGR. However, the EDC conjugate of ANTS-midi-GAGR was not precipitated by 75% ethanol, suggesting that the EDC conjugate cannot be purified using 75% ethanol.)

## 5 Measurement of the amount of ANTS-polysaccharide that enters the brain and blood circulation

### 5.1 Administration and measurement of ANTS-midi-GAGR

To examine the BBB-permeability of ANTS-midi-GAGR, 40 μL of 1 mM ANTS-midi-GAGR (40 nmole of ANTS-midi-GAGR into 15 mL total blood volume per rat = 2.67 μM in the blood circulation of a rat) was administered into the nostril of Sprague-Dawley rats (Female, 250–320 g, age of 8–10 weeks).

1. Rats were quickly anesthetized in an isoflurane induction chamber (isoflurane from Henry Schein Animal Health (Dublin, OH)).
2. 5% isoflurane is administered into animal by a vaporizer with oxygen flowmeter (0.8–1.5 L/min). The percent of isoflurane was later adjusted to 2% until animal loses righting reflex.
3. 20 μL of 1 mM ANTS-midi-GAGR was intra-nasally administered to each nostril.
4. Animals were kept in the anesthetized condition for 5 min after the administration of ANTS-polysaccharide to prevent the squirting-out of ANTS-polysaccharide from the noses.
5. At 1, 6 and 24 h after the administration of ANTS-polysaccharide, animals were sacrificed using a guillotine.
6. About 1 mL of trunk blood (6 h post-administration) was collected in a vial immediately after decapitation.
7. Trunk blood was incubated at room temperature for 1 h to coagulate and centrifuged at 3000 × *g* for 10 min to remove the coagulated, which yields ∼400 μL of serum.
8. Simultaneously, the olfactory bulb tract and whole brain were also dissected out of the head of the decapitated animal.
9. The brain was further dissected to the frontal, parietal, temporal, and occipital cortices, striatum, hypothalamus/thalamus, cerebellum, brainstem, and hippocampus.
10. Each brain parts and olfactory bulb tract were washed with 0.9% saline and homogenized in the equivalent volume of 1× PMEE buffer (pH 7.0; 35 mM KOH, 35 mM PIPES, 5 mM MgSO4, 1 mM EGTA, 1% BSA, and 0.5 mM EDTA) containing 1% Igepal CA-630 using a glass homogenizer (Wheaton, Millville, NJ).
11. The homogenized brain extract was centrifuged at 14,500 × *g* for 20 min and at 100,000 × *g* for 30 min to obtain brain cytosol.
12. The amounts of ANTS-polysaccharide in the serum and the cytosols extracted from olfactory bulb and brain were measured by fluorometry.

### 5.2 Quantification of ANTS-midi-GAGR in brain tissues and blood

We quantified the amounts of ANTS-midi-GAGR in the samples using the standard curve of ANTS and the conjugation ratio of 10:1 for ANTS to midi-GAGR (Fig. 1).

**Fig. 2:**
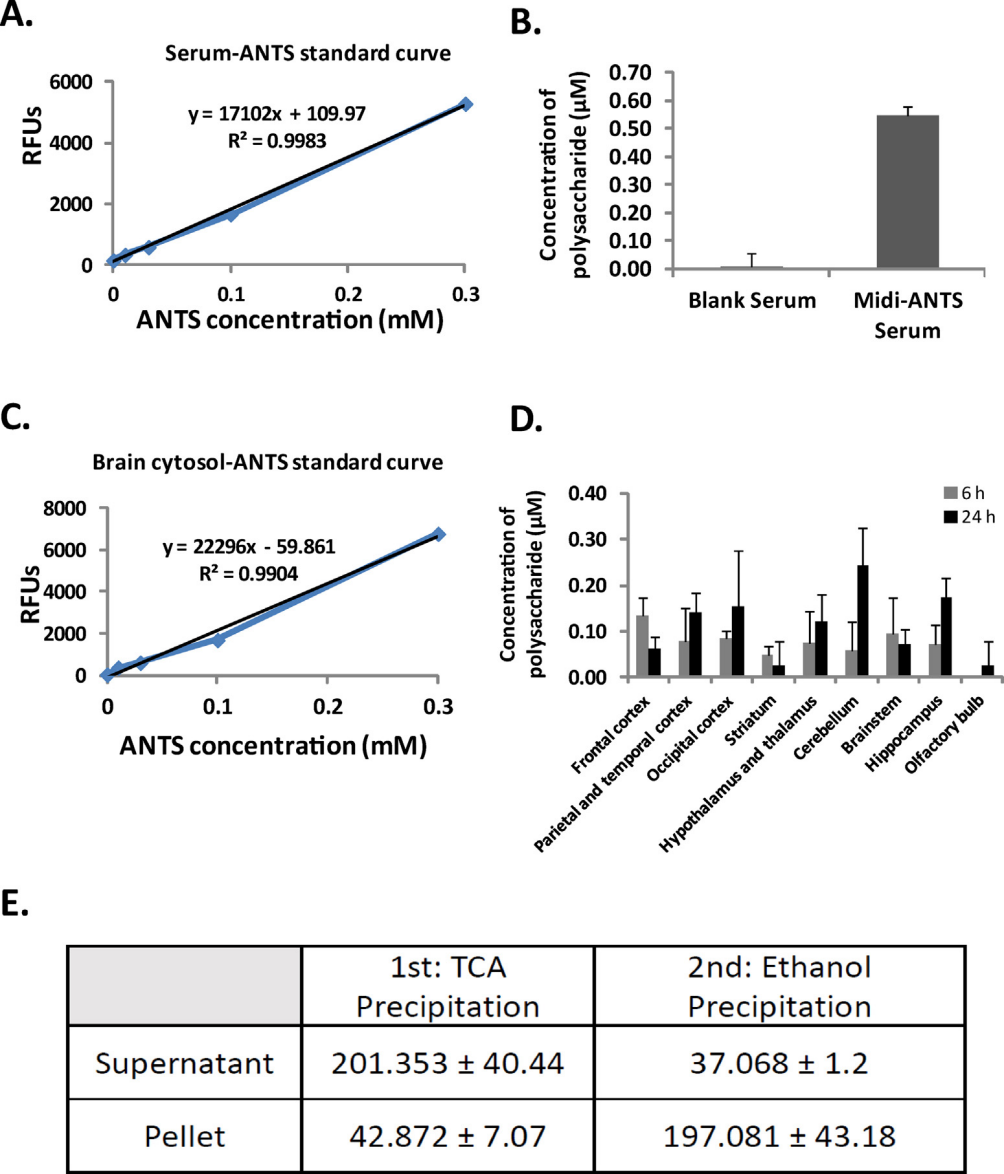
(A) The standard curve of emission at 520 nm of ANTS in the sera at 0, 0.01, 0.03, 0.1 and 0.3 mM (*n* = 3). (B) The calculated concentrations (Mean ± Std. Error) of midi-GAGR in the sera at 6 h after the intranasal administration of ANTS-midi-GAGR (*n* = 3). (C) The standard curve of emission at 520 nm of ANTS in the brain cytosol at 0, 0.01, 0.03, 0.1, and 0.3 mM (*n* = 3). (D) The calculated concentrations (Mean ± Std. Error) of midi-GAGR in the different brain regions at 6 and 24 h after the intranasal administration of ANTS-midi-GAGR (*n* = 3). (E) The RFUs (Mean ± Mean ± Std. Error) of the supernatants and pellets of TCA precipitation and ethanol precipitation of sera (*n* = 5).

1. The RFUs of the cytosols extracted from the olfactory bulb tracts of all rats were lower than ∼50 RFUs that were the same as the basal fluorescence units of those of rats administered with saline alone (data not shown). ANTS-midi-GAGR does not appear to be accumulated in the olfactory bulb tract or passes the tract very quickly.
2. The RFUs of the sera and brain cytosols were significantly above 50 RFUs. The RFU values of brain cytosols and sera were converted to the concentrations of midi-GAGR using the ratio of 10:1 for ANTS to midi-GAGR.
3. Since we found that the RFUs of ANTS in the serum and brain cytosol were higher than those of an equivalent concentration of ANTS in water, we generated new standard curves using 0, 0.01, 0.03, 0.1, and 0.3 mM ANTS in the brain cytosol and serum.
4. Based on the standard curve of ANTS in the serum (Fig. 2A), the concentration of midi-GAGR in serum was 0.55 ± 0.03 μM (Mean ± Std. Error, Fig. 2B).
5. Based on the standard curve of ANTS in the brain cytosol (Fig. 2C), the concentrations of midi-GAGR in different brain parts are shown in Fig. 2D. The concentrations of midi-GAGR were increased significantly in different brain regions after 6 h. The increased concentrations of midi-GAGR were maintained until 24 h. On the other hand, we could not detect ANTS-midi-GAGR in those brain regions at 1 h after nasal spray. Thus, ANTS-midi-GAGR after one-time intra-nasal administration enters the brain within 6 h and remain high until 24 h.

(*Note*: we have tried to use a confocal microscope available onsite to visualize the distribution of ANTS-midi-GAGR on brain slices but could not because our current microscope is not equipped with the filter set for the excitation wavelength (350 nm) for ANTS.)

### 5.3 Examination of the structural intactness of ANTS-midi-GAGR in the serum

It was possible that the fluorescence of the sera was emitted from free ANTS that might be generated by the cleavage of ANTS-midi-GAGR during the circulation in the blood. Therefore, we examined whether ANTS-midi-GAGR in the sera was structurally intact or not by 75% ethanol precipitation that only precipitate ANTS-midi-GAGR but not free ANTS.

1. 100 μL of the supernatant was added with 300 μL of 100% ethanol to make the final concentration of 75% ethanol and centrifuged at 3000 × *g* to precipitate serum polysaccharides including ANTS-midi-GAGR, leaving polysaccharide-free ANTS in the supernatant.
2. The pellet was resuspended in the equivalent volume of water to that of the supernatant.
3. The RFUs of the supernatant and pellet resuspension were measured by fluorometry.
4. The RFUs of the supernatant fell down to below 50 while those of the pellet resuspension were close to those of the supernatant before ethanol precipitation. This suggests that most of ANTS-midi-GAGR was structurally intact in the serum.

### 5.4 Examination of the binding of ANTS-midi-GAGR to serum protein

We also examined whether ANTS-midi-GAGR in the serum bound to serum proteins like albumin or not. We used TCA that should precipitate all the proteins and protein-bound molecules including polysaccharides but leave protein-free polysaccharides in the supernatant.

1. 100 μL of serum sample in a microtube was added with 10 μL of TCA to make the final concentration of 10% TCA, incubated at 4 °C for 10 min, and centrifuged at 15,700 ×* g* for 5 min.
2. The supernatant over the pellet was transferred to a new tube and the pellet containing proteins was resuspended in 100 μL of water.
3. The RFU of the pellet resuspension was measured by fluorometry.
4. The RFUs of the pellet resuspension was below the basal fluorescent units (50 RFUs) while those of the supernatants still yielded ∼201 RFUs (Fig. 2C). It suggests that little ANTS-polysaccharide was precipitated along with serum proteins.
5. 100 μL of the supernatant was added with 300 μL of 100% ethanol to make the final concentration of 75% ethanol and centrifuged at 3000 × *g* to precipitate serum polysaccharides including ANTS-midi-GAGR.
6. The pellet was resuspended in 50 μL water.
7. The RFUs of the supernatant and pellet resuspension were measured by fluorometry.
8. The RFU of the supernatant was below the basal fluorescent units (50 RFUs) while the pellet resuspension yielded ∼201 RFUs, suggesting that most ANTS-midi-GAGR remained intact in the supernatant after TCA precipitation.

(*Note*: ANTS-midi-GAGR that enters the brain and blood circulation does maintain its intact structure inside animal and does not bind to serum protein for 6 h after its intra-nasal administration. Given that the cerebrospinal fluid should contain less digestive enzymes than the peripheral blood, we speculated that ANTS-midi-GAGR in the brain should be intact as well.)

## 6 Additional information

### 6.1 Background

Polysaccharides are used for a variety of medical applications because of their advantageous properties such as non-inflammatory, long half-life, and few side-effects in humans. Generally, polysaccharides are assumed to have no physiological effect, thus being considered as an optimal supportive material for medical applications. Recently, some polysaccharides showed therapeutic potentials for the treatment of neurodegenerative diseases [4], [5], [6], [7], [8], [9]. Some appear to elicit responses similarly to an endogenous polysaccharide, polysialic acid (PSA) that enhances neurogenesis in neurons [10], [11]. However, lack of the information about the BBB-permeability of those polysaccharides has limited animal study to examine the efficacy of the polysaccharides in treating a neurological disorder. A radiolabel can be used to tag and track a polysaccharide in animal. However, radiolabeling is not ideal for animal study because radioactivity potentially damages animal tissues and increases the tedious workload for animal husbandry. In contrast, ANTS is a harmless fluorophore that can be easily conjugated to polysaccharide and detected by fluorometry. Moreover, since ANTS is highly soluble in 75% ethanol but polysaccharides are not, ANTS-polysaccharide conjugates can be easily separated from free ANTS using 75% ethanol. Our simple method to label, track, and quantify a target polysaccharide inside animal should provide a useful tool to the future study of neurotrophic polysaccharides in animal.
